# A Soft Amphibious Voxel-Type Quadruped Robot Based on Origami Flexiball of Rhombic Dodecahedron

**DOI:** 10.3390/biomimetics9080482

**Published:** 2024-08-09

**Authors:** Fuwen Hu, Yanqiang Li

**Affiliations:** School of Mechanical and Material Engineering, North China University of Technology, Beijing 100144, China

**Keywords:** origami, soft robot, amphibious robot, underwater robot, modular robot, quadruped robot, voxel-type robot

## Abstract

The research work presents a novel voxel-type soft amphibious robot based on an assembly of origami flexiballs. The geometric and elastic constitutive models of the origami flexiball are theoretically established to elucidate its intricate deformation mechanism. Especially, the zero-energy storage phenomenon and the quasi-zero-stiffness characteristic are revealed to prove that the origami flexiball is suitable for serving as soft robotic components. As a proof of concept, fourteen origami flexiballs are interconnected to form a quadruped robot capable of walking or crawling in both underwater and terrestrial environments, including flat surfaces and sandy terrain. Its adaptability across multiple environments is enhanced by the origami polyhedra-inspired hollow structure, which naturally adjusts to underwater conditions such as hydrostatic pressure and currents, improving stability and performance. Other advantages of the voxel-type soft amphibious quadruped robot include its ease of manufacture using 3D printing with accessible soft elastic materials, ensuring rapid and cost-effective fabrication. We anticipate its potentially versatile applications, including underwater pipeline inspections, offshore maintenance, seabed exploration, ecological monitoring, and marine sample collection. By leveraging metamaterial features embodied in the origami polyhedra, the presented voxel-type soft robot exemplifies an innovative approach to achieving complex functionalities in soft robotics.

## 1. Introduction

Amphibious robots, characterized by their trans-medial integration of terrestrial and aquatic functionalities, represent a burgeoning field poised at the intersection of robotics, biomechanics, and environmental science. These dynamic platforms exhibit the capability to navigate through a spectrum of complex environments, spanning marshlands, swamps, and littoral regions, underscoring their pivotal role in a gamut of missions ranging from marine exploration to search and rescue operations, environmental preservation endeavors and military reconnaissance initiatives. As such, scholars advocate for an embrace of biomimicry, leveraging the refined designs honed by millennia of evolutionary selection in nature. Amphibious organisms, such as frogs [1], lobster [2], crabs [3], turtles [4], beavers [5], cormorants [6], snakes [7], and salamanders [8], stand as exemplars of superior trans-medial locomotion capabilities, offering invaluable insights for biomimetic emulation. However, no matter whether it is imitating a certain organism or leveraging a hybrid and multimechanism locomotion, reduplicating the refined designs honed by millennia of evolutionary selection in nature to achieve amphibious biomimetic robots also remains elusive, such as energy usage efficiency, compact body structures, excellent motion ability, high adaptability to natural environments, and embodied intelligence [9].

More recently, origami-based solutions have intensively demonstrated powerful potential in the burgeoning domain of soft robotics. Various origami-based locomotion modes, including crawling [10], walking [11], swimming [12], jumping [13], wheeling [14], flying [15], and multimodal locomotion [16], offer a distinct pathway departing from conventional biomimetic approaches to achieve diverse and anticipated robotic movements. For instance, an interesting origami polyhedron, commonly called an origami flexiball, was introduced into soft robotics by our research team [17]. This compact origami model has shape-switchable compliance with multiple degrees of freedom and is capable of synchronous hierarchical shape-shifting. The monolithic deformations of the polyhedron are driven by the second-order deformations of built-in rhombic meshes, or vice versa. Additionally, by translational copies of themselves, the soft origami polyhedra can be assembled into more complex reconfigurable assemblages. These metamaterial or metastructure features embodied in the origami polyhedra voxel have empowered the creation of various soft robots. Furthermore, a kind of origami polyhedra-based soft multicellular robot with multimodal locomotion has been demonstrated [18]. In particular, the in-line three voxels configuration manifests really amazing adaptability: crawling on the ground and horizontally and vertically climbing pole-like structures without the need to reorganize its pattern or reshape its morph. In another research, it serves as a soft robotic body to replicate jellyfish-like jet-propelled swimming [19].

Undoubtedly, thanks to the minimalist architecture of the origami flexiball model, it has permeated into many directions of soft robotics, such as soft modular robots, soft origami robots, soft biomimetic robots, soft voxel-based robots, as well as soft printable robots. In this latest work, a novel metamaterial or metastructure feature embodied in the origami flexiball is theoretically revealed for the first time, that is, the zero-energy storage phenomenon and the quasi-zero-stiffness characteristic, which makes it suitable for flexible robotic components. Further, as an interesting proof of concept, the origami polyhedra manifests a novel possibility to form a voxel-type soft amphibious quadruped robot, which can achieve aquatic-terrestrial walking and crawling without the extra shape-shifting or the reconfigurable method. Many current amphibious robots employ two distinct locomotion systems that require manual switching between modes for moving in water and terrestrial movement. This manual switching reduces their efficiency and reliability. However, the proposed approach addresses the long-standing challenge of developing an amphibious robot capable of moving seamlessly in both aquatic environments and on land, utilizing a single mechanism [20]. In addition, owing to the fact that the origami polyhedra is essentially hollow, the presented voxel-type soft amphibious robot can naturally adapt to surge and hydrostatic pressure in deep water or deep-sea environment, especially in the littoral zone (the transition between water and land). Potentially, this promises that it can be used for underwater pipeline inspections, offshore infrastructure maintenance, seabed exploration, sample collection from marine environments, and ecological aquatic phenomena monitoring and data collection [21].

## 2. Geometry and Elastic Constitutive Relation of Origami Flexiball

### 2.1. Geometry of Origami Flexiball

As a popular hand-made origami model, the origami flexiball is constructed by folding and snapping modular sheet units into a flexible polyhedron with extruded outward faces. In geometry, these rhombic polyhedra have many types of symmetries because they are convex, face-transitive (isohedral), and isotoxal (edge-transitive). Here, p denotes the edge length of the rhombic planes, q denotes the extruded height of the rhombic planes, α represents the acute angle in the rhombic planes, β represents the obtuse angle in the rhombic planes. Initially, the acute and obtuse angles of the congruent rhombic planes are 70.5° and 109.5°, respectively. If γ represents the dihedral angle between two adjacent rhombic planes, as for the symmetries, totally there are three types of dihedral angles, denoted by $\gamma_{1}$, $\gamma_{2}$, and $\gamma_{3}$, respectively. Initially, their values are 60°. Furthermore, $L_{0}$ and $L_{1}$ represent the lengths of the two diagonals of the rhombic surfaces, and h represents the nominal height between the two top rhombic plane and its symmetrical rhombic plane.

Geometrically, the following formulas can be deduced:

(1) $$\left\{\begin{array}{c} h=2q+L_{1}\;\;\;\;\;\;\;\;\;\\ L_{0}=\sqrt{{4p}^{2}-{L_{1}}^{2}} \end{array}\right.\;.$$

**Figure 1:**
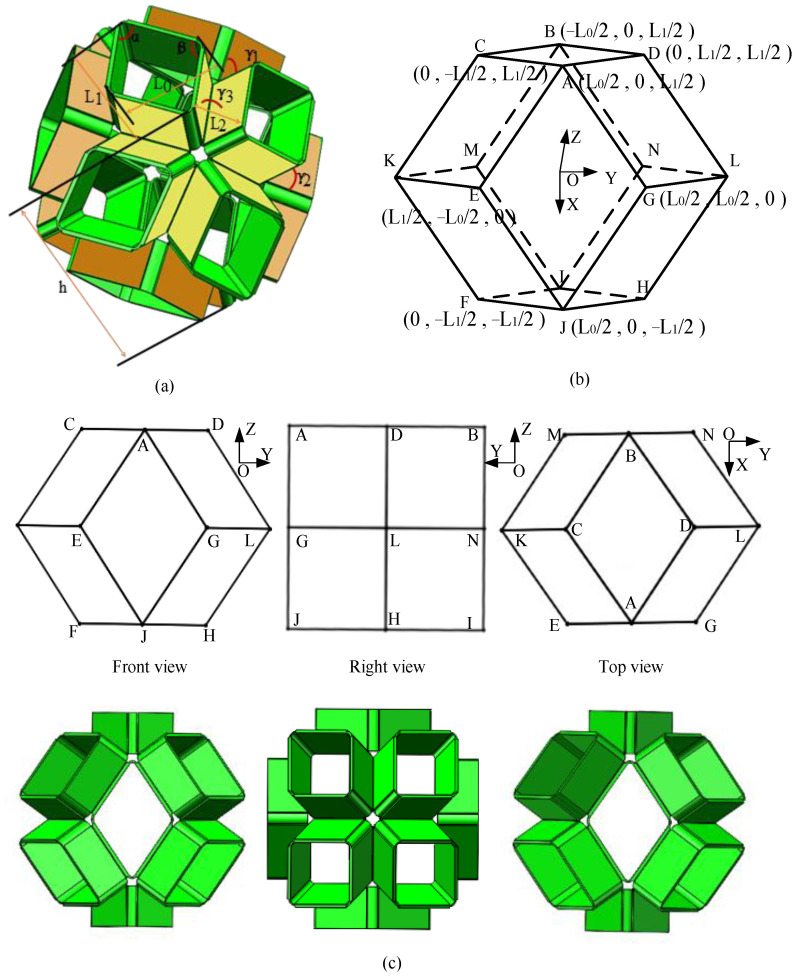
Geometry of rhombic dodecahedron origami flexiball. (**a**) Geometric parameters of rhombic dodecahedron origami flexiball; (**b**) vertices coordinates of the rhombic dodecahedron; (**c**) symmetry and projection view of rhombic dodecahedron origami flexiball.

If we establish a Cartesian coordinate system at the centroid of the rhombic dodecahedron, as illustrated in Figure 1b, the Z and X axes are perpendicular and normal to the top and front surfaces of the rhombic dodecahedron in the outward direction, respectively. Due to symmetry in the Z and X directions, the top and front views of the unit cell are identical. Then, the coordinate positions of the vertices can be derived, such as $A(\frac{1}{2}L_{0},0,\frac{1}{2}L_{1})$, $B(-\frac{1}{2}L_{0},\;0,\frac{1}{2}L_{1})$, $C(0,-\frac{1}{2}L_{1},\frac{1}{2}L_{1})$, $D(0,\frac{1}{2}L_{1},\frac{1}{2}L_{1})$, $E(\frac{1}{2}L_{1},-\frac{1}{2}L_{0},0)$, $F(0,-\frac{1}{2}L_{1},-\frac{1}{2}L_{1})$, $G(\frac{1}{2}L_{0},\frac{1}{2}L_{0},0)$, and $J(\frac{1}{2}L_{0},0,-\frac{1}{2}L_{1})$. Further, the vectors of the rhombic dodecahedron edges can be determined using

(2) $$\left\{\begin{array}{l} \vec{CA}=(\frac{1}{2}L_{0},\;\frac{1}{2}L_{1},\;0)\\ \vec{DA}=(\frac{1}{2}L_{0},-\frac{1}{2}L_{1},\;0)\\ \vec{EA}=\{\frac{1}{2}(L_{0}-L_{1}),\;\frac{1}{2}L_{0},\;\frac{1}{2}L_{1}\}\\ \vec{GA}=(0,-\frac{1}{2}L_{0},\;\frac{1}{2}L_{1})\\ \vec{EJ}=\{\frac{1}{2}(L_{0}-L_{1}),\;\frac{1}{2}L_{0},-\frac{1}{2}L_{1}\}\\ \vec{FJ}=(\frac{1}{2}L_{0},\;\frac{1}{2}L_{1},\;0) \end{array}\right..$$

As a further step, the normal vectors of the extruded edges of the rhombic planes can be calculated using

(3) $$\left\{\begin{array}{l} \vec{m_{1}}=\vec{DA}\times \vec{CA}=(0,\;0,\;\frac{1}{2}{L_{0}L}_{1})\\ \vec{m_{2}}=\vec{CA}\times \vec{EA}=\{\frac{1}{4}{L_{1}}^{2},-\frac{1}{4}L_{0}L_{1},\;\frac{1}{4}({{{L_{0}}^{2}+L}_{1}}^{2}-L_{0}L_{1})\}\\ \vec{m_{3}}=\vec{EA}\times \vec{GA}=\{\frac{1}{2}L_{0}L_{1},\;\frac{1}{4}({L_{1}}^{2}-L_{0}L_{1}),\;\frac{1}{4}(L_{0}L_{1}-{L_{0}}^{2})\}\\ \vec{m_{4}}=\vec{EJ}\times \vec{FJ}=\{\frac{1}{4}{L_{1}}^{2},-\frac{1}{4}L_{0}L_{1},\;\frac{1}{4}({{{{-L}_{0}}^{2}-L}_{1}}^{2}+L_{0}L_{1})\} \end{array}\right..$$

Then, the angles between the extruded edges of the rhombic planes, $\gamma_{1}$, $\gamma_{2}$, and $\gamma_{3}$, can be calculated using

(4) $$\left\{\begin{array}{c} \gamma_{1}=\cos^{-1}<\vec{m_{1}},\vec{m_{2}}>{=\cos}^{-1}\left\{\begin{array}{c} ({L_{0}}^{3}L_{1}-{L_{0}}^{2}{L_{1}}^{2}+{L_{1}}^{3}L_{0})/\\ \;(L_{0}L_{1}\times \sqrt{{L_{0}}^{4}+2{L_{1}}^{4}-2{L_{0}}^{3}L_{1}-2{L_{1}}^{3}L_{0}+4{L_{0}}^{2}{L_{1}}^{2}}) \end{array}\right\}\;\;\;\;\;\;\\ \gamma_{2}=\cos^{-1}<\vec{m_{2}},\vec{m_{3}}>{=\cos}^{-1}\left\{\begin{array}{c} (2{L_{0}}^{3}L_{1}-{L_{0}}^{2}{L_{1}}^{2}+2L_{0}{L_{1}}^{3}-{L_{0}}^{4})/\\ (\sqrt{{L_{0}}^{4}+2{L_{1}}^{4}-2{L_{0}}^{3}L_{1}-2{L_{1}}^{3}L_{0}+4{L_{0}}^{2}{L_{1}}^{2}}\times \\ \sqrt{{L_{0}}^{4}+{L_{1}}^{4}+6{L_{0}}^{2}{L_{1}}^{2}-2{L_{0}}^{3}L_{1}-2{L_{1}}^{3}L_{0}}) \end{array}\right\}\;\;\;\;\;\;\;\;\;\;\;\;\;\;\;\;\;\;\;\;\\ \gamma_{3}=\cos^{-1}<\vec{m_{2}},\vec{m_{4}}>{=\cos}^{-1}\left\{\begin{array}{c} (2{L_{0}}^{3}L_{1}-2{L_{0}}^{2}{L_{1}}^{2}+2{L_{1}}^{3}L_{0}-{L_{0}}^{4})/\\ (\sqrt{{L_{0}}^{4}+2{L_{1}}^{4}-2{L_{0}}^{3}L_{1}-2{L_{1}}^{3}L_{0}+4{L_{0}}^{2}{L_{1}}^{2}}\times \\ \sqrt{{L_{0}}^{4}+2{L_{1}}^{4}+4{L_{0}}^{2}{L_{1}}^{2}-2{L_{0}}^{3}L_{1}-2{L_{1}}^{3}L_{0}}) \end{array}\right\}\;\;\;\;\;\;\;\;\;\;\;\;\;\;\;\;\;\;\;\; \end{array}\right..$$

Obviously, the formulas established above reveal the intrinsic geometry of the origami flexiball model. Specifically, they can depict the complex deformation depending upon one dependent variable: the nominal height h of the origami flexiball model.

### 2.2. Elastic Constitutive Relation of Origami Flexiball

Further, the great motion mobility or the shape-shifting ability of the origami flexiball can make it absorb elastic energy of multiple magnitudes. It is assumed that the paper faces remain rigid during deformations; that is, they do not store any energy. Then, we only estimate the fold elastic energy $U_{crease}$ of one crease by parameterizing its torsional stiffness k, the rest crease angle $\gamma_{0}$, and the opening angle $\gamma_{i\;}$ [22,23]. Further, the total elastic energy $U_{total}$ of the whole origami flexiball can be approximated using

(5) $$U_{total}=\sum_{i=1}^{24}U_{crease-i}=\frac{1}{2}\sum_{i=1}^{24}k(\gamma_{i}-\gamma_{0}{)}^{2}.$$

It should be noted that the rhombic dodecahedron is not homogeneous symmetrical. As illustrated in Figure 1, the rhombic dodecahedron has two types of vertices: eight vertices with three edges and six vertices with four edges. This feature of the spatial geometric structure determines that the elastic constitutive model of the origami flexiball has obvious directivity. Due to symmetry, the mechanical properties in the Z and X directions are identical, while the mechanical properties in the Y direction are different. This implies that the torsional spring stiffness k per unit length of the crease has obvious directionality. Here, in the Z and X directions, k is marked as $k_{z/x}$, and in the Y direction, k is marked as $k_{y}$. In another aspect, when the origami flexiball of the rhombic dodecahedron is compressed in the Z and X directions, according to the symmetry and the positions of edges, the closing or opening dynamics of the 24 crease angles $\gamma_{i}$ are divided into three kinds of variation, which are marked as $\gamma_{z/x-1}$, $\gamma_{z/x-2}$, and $\gamma_{z/x-3}$, each with eight crease angles. In contrast, when the origami flexiball of the rhombic dodecahedron is compressed in the Y direction, according to the symmetry and the positions of edges, the closing or opening dynamics of the 24 crease angles $\gamma_{i}$ are divided into two kinds of variation, which are marked as $\gamma_{y-1}$ and $\gamma_{y-2}$, with 16 and 8 crease angles, respectively. Consequently, the total elastic energy with directional variables can be expressed using

(6) $$\left\{\begin{array}{l} \;U_{z/x}=\frac{1}{2}\;k_{z/x}\;\;(8\sum_{i=1}^{3}{\left(\gamma_{z/x-i}-\gamma_{0}\right)}^{2}),\;i=1,2,3\\ U_{y}={\frac{1}{2}\;k_{y}(16(\gamma_{y-1}-\gamma_{0})}^{2}+8{\left(\gamma_{y-2}-\gamma_{0}\right)}^{2}) \end{array}\right..$$

According to Castigliano’s first theorem, the partial derivative of the strain energy with respect to any generalized displacement equals the corresponding generalized force. This principle is applicable to both linear and nonlinear elastic bodies. By taking the derivative of the potential $U_{total}$ with respect to the nominal height h, the force-displacement relation can be obtained as

(7) $$F=\frac{dU_{total}}{dh}=k\sum_{i=1}^{24}\left(\gamma_{i}-\gamma_{0})(\frac{d\gamma_{i}}{dh}\right).$$

**Figure 2:**
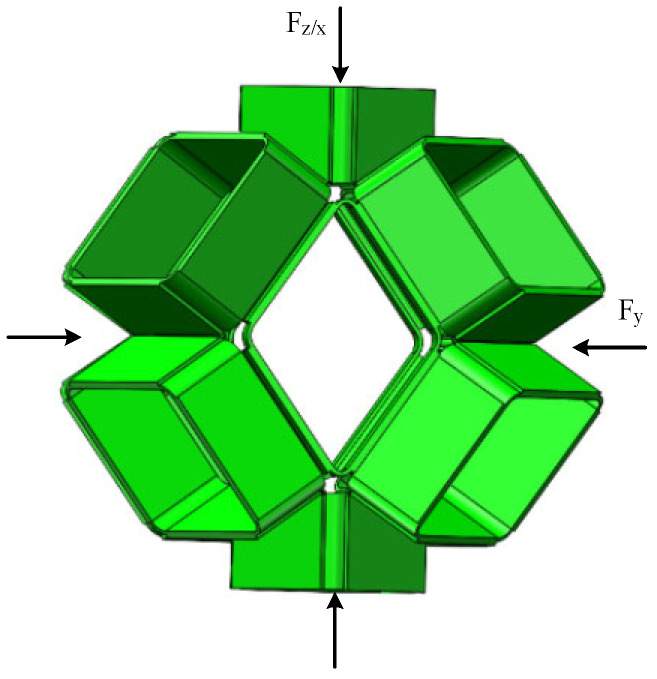
Schematic of a rhombic dodecahedron origami flexiball under uniaxial compression in the Z or X directions and in the Y direction.

As illustrated in Figure 2, taking into account the anisotropy of direction, we can obtain the force-displacement relations in the Z or X directions, and in the Y direction, they can be further expressed using

(8) $$\left\{\begin{array}{l} F_{z/x}=\frac{dU_{z/x}}{dh}=k_{z/x}\;\;\left(8\sum_{i=1}^{3}\left(\gamma_{\frac{z}{x}-i}-\gamma_{0}\right)\left(\frac{d\gamma_{\frac{z}{x}-i}}{dh}\right)\right),\;i=1,\;2,\;3\\ F_{y}=\frac{dU_{y}}{dh}=k_{y}\;\;(16(\gamma_{y-1}-\gamma_{0})(\frac{d\gamma_{y-1}}{dh})+8(\gamma_{y-2}-\gamma_{0})(\frac{d\gamma_{y-2}}{dh}))\; \end{array}\right..$$

For the testing sample, the rhombic dodecahedron model has a rhombic plane edge length p of 15.41 mm and an extruded height q of 9.72 mm for its rhombic surfaces. The 3D printable material used to fabricate the printable origami flexiball is thermoplastic polyurethane (TPU), and the basic thickness of the rhombic surfaces is 1.47 mm. According to the force-displacement relation in Equation (8), compression tests on the sample were conducted to identify the torsional spring stiffness $k_{z/x}$ of the crease as 4.2057 $N\cdot {rad}^{-1}\cdot {mm}^{-1}$, and the torsional spring stiffness $k_{z/x}$ in the direction Y as 2.6547 $N\cdot {rad}^{-1}\cdot {mm}^{-1}$.

**Figure 3:**
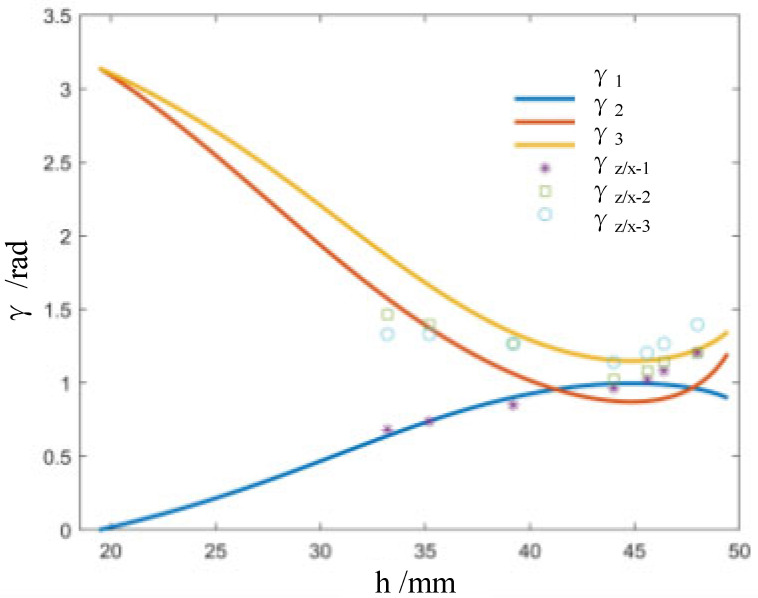
Theoretically and actually changing curves of the opening creases angles versus the nominal height.

Further, according to Equation (4), following the changes of the nominal height h, we compared the theoretically changing values with the actually changing values of $\gamma_{z/x-1}$, $\gamma_{z/x-2}$, and $\gamma_{z/x-3}$. As illustrated in Figure 3, the relationship between the opening crease angles and the nominal height is not linear, in particular, during the early elastic deformation stage. Further, the change trends of theoretical values and measured values of opening angles of the creases are basically the same, and their values are very close.

**Figure 4:**
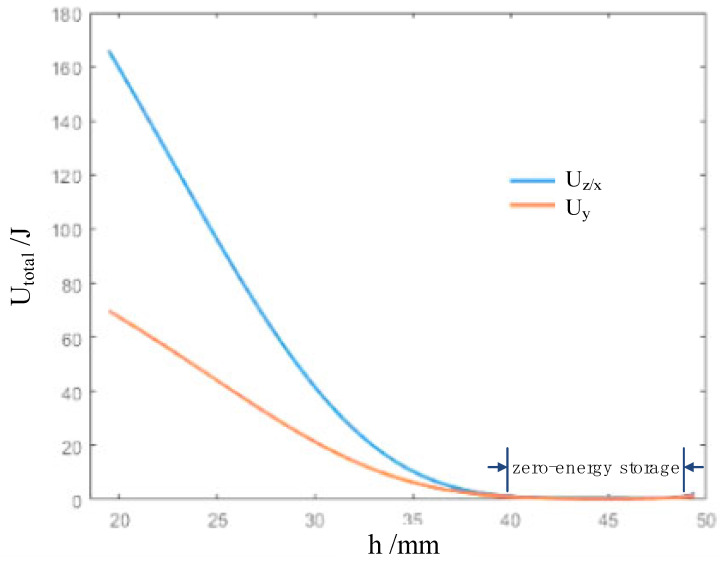
Theoretically changing curves of the elastic potential energy versus the nominal height.

According to Equation (6), we further study the relationship between the elastic potential energy of the cell and the nominal height h of the cell. As can be seen from Figure 4, when the height is 35 mm as the critical point, the elastic energy changes rapidly and then changes slowly. This means that there is an obvious nonlinear elastic stage before 35 mm, showing a clear zero-energy mode.

**Figure 5:**
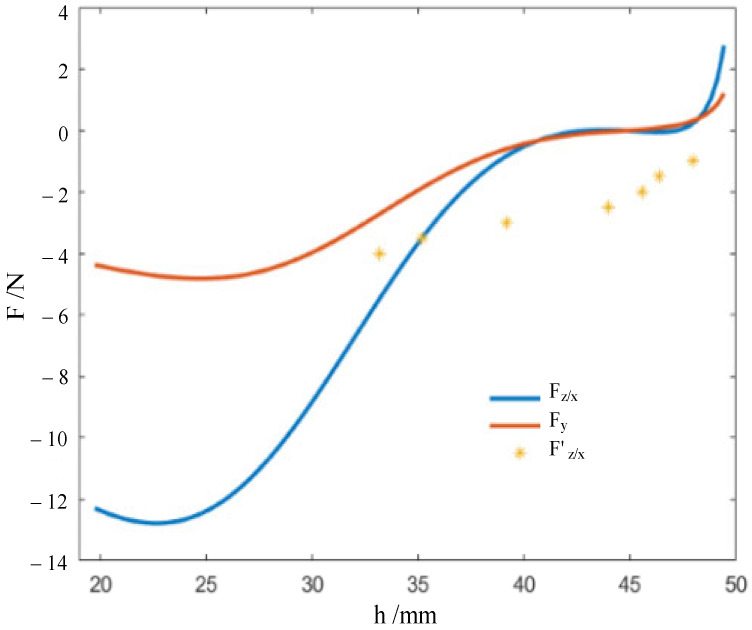
Theoretically and actually changing curves of forces versus the nominal height.

Further, according to Equation (8), we quantitatively studied the force-displacement relation of the real origami flexiball, as illustrated in Figure 5. The transition points of the force-displacement curve and the energy-displacement curve are basically close. Currently, we observe that there is a big deviation between the theoretical value of the force and the actual applied value. The reason behind it is that the theoretical value of the force in Equation (8) is based on Castigliano’s first theorem, while in the experimental work, the experimental force values are loaded step by step, and the corresponding deformation displacements are measured. That is, the experimental work seems to follow Castigliano’s second theorem (a partial derivative of strain energy with respect to applied forces), which is only applicable to linear elastic materials. Under the assumption of small displacements, that is, in the linear elastic stroke, the theoretical and experimental values should keep a small deviation. Therefore, the large deviation between them may prove that the defamation of the origami flexiball has strong nonlinearity. This type of nonlinearity is largely due to the topological structure of the origami flexiball. As shown in Figure 4, the zero-energy storage phenomenon is observed. Further, Figure 5 shows that in the initial stage of deformation, the origami flexiball exhibits the quasi-zero-stiffness characteristic. To the best of our knowledge, this novel metamaterial feature of quasi-zero-stiffness characteristic embodied in the origami flexiball is revealed for the first time. This interesting characteristic enables the origami flexiball to absorb shocks and impacts more effectively, making it suitable for applications requiring robust shock absorption, such as in protective gear, impact-resistant packaging, or flexible robotic components. The demonstrated soft robot fully composed of this will reveal good adaptability to uneven terrain and experimental settings.

## 3. Soft Amphibious Robot Based on Origami Flexiball

### 3.1. Configuration of the Soft Voxel-Based Robot

**Figure 6:**
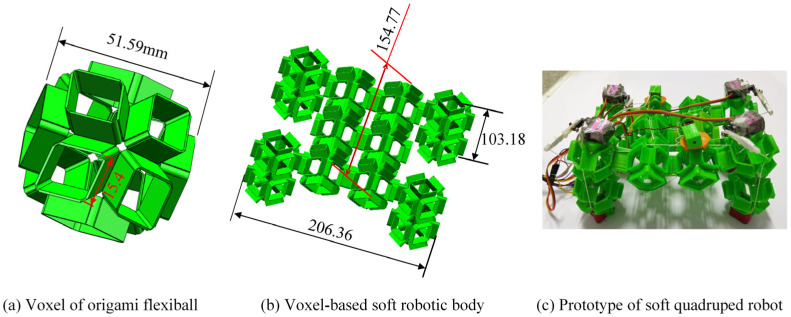
Overall structure of origami flexiball-based soft amphibious quadruped robot.

As stated previously, many attractive attributes of the origami flexiball empower it to be a promising type for use as a soft robotic voxel or robotic genetic cell (see Figure 6a). In this work, fourteen origami flexiballs are connected into an assemblage to mimic the basic form of a quadruped robot, as illustrated in Figure 6b. Owing to the fact that thermoplastic polyurethane (TPU) is a linear segmented block polymer with excellent mechanical properties of large elongation and moderate tensile and compressive strength, herein, it is adopted as the feedstock filaments to fabricate the printable origami flexiball. The finalized prototype is shown in Figure 1c. Considering that the rope-motor-driven strategy has high flexibility, a high strength-to-weight ratio, and a possible small bending radius to suit the size and strength of the proposed origami structure, the rope-motor-driven method is employed and consists of an SG90 servo motor, a servo holder, and ropes. Basically, the elongation and friction coefficient of the synthetic ropes are supposed to be zero, and the power of the servo motor is transferred into the elastic deformation energy of the soft dodecahedron. The SG90 servo motor has a stall torque of 1.4 kg/cm when given a voltage input of 4.8 volts. The energy supply of the soft robot is rechargeable lithium battery.

### 3.2. Locomotion Modes of the Soft Voxel-Based Robot

Legged amphibious robots are an early type of amphibious biomimetic robot inspired by arthropods such as crabs, lobsters, and newts. They emulate the rhythmic leg movements of these organisms, enabling them to adapt to complex aquatic and terrestrial environments. Basically, gait refers to a prescribed sequence of leg joint configurations executed during locomotion. Quadruped robots exhibit two principal forms of gait: static and dynamic. Static gait modes, such as crawling or waving, necessitate that the vertical projection of the robot’s center of gravity consistently resides within the polygon defined by its supporting limbs to ensure stable movement. In contrast, dynamic gaits, including trotting, pacing, or galloping, allow the robot’s center of gravity to intermittently project outside this support polygon, facilitating dynamic stability. As a representative dynamic gait, the trot gait involves a coordinated locomotive pattern where each diagonal pair of the robot’s feet alternates between synchronous lifting, thrusting forward, and grounding. This rhythmic sequence enables effective forward progression while maintaining stability and optimizing energy efficiency. The choice between static and dynamic gaits depends on specific locomotive requirements, considering factors such as stability maintenance, terrain adaptability, and energy efficiency.

**Figure 7:**
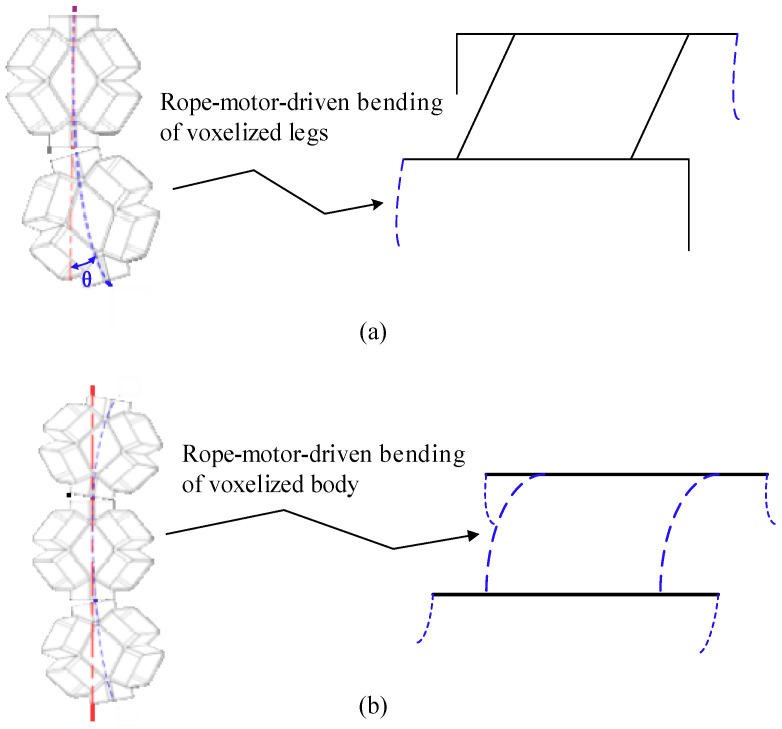
Locomotion modes of the origami flexiball-based soft amphibious robot. (**a**) Flexible bending of the legs to generate the walking gait; (**b**) flexible bending of the legs and body to generate the crawling gait.

For the presented soft voxel-based robot, we generated two modes of locomotion: trot-like walking and body-down-to-ground crawling. As illustrated in Figure 7, the rope-motor-driven bending of the voxelized legs makes it possible to perform a trot gait by alternating the stance and swing phases of each pair of legs (diagonal gait). Owing to the full softness of the soft voxel-based robot, the voxelized body can also bend down to the terrain to achieve the locomotion mode of crawling. Having a lower center of mass, the sprawled posture is generally more stable than the fully upright orientation of the legs. The locomotion modes easily change from walking to crawling and vice versa. Undoubtedly, the multimodal locomotion empowers the soft voxel-based robot with good adaptability to complex terrains.

Assuming for simplicity that the soft voxelized leg is accurately controlled by the bending angle θ and further referring to the control method [24], a closed-loop dynamic controller for a soft voxelized leg can be introduced as follows:

(9) $$I(\theta )(\ddot{\theta }-\ddot{\bar{\theta }})+C(\theta ,\dot{\theta })(\dot{\theta }-\dot{\bar{\theta }})-\tau_{d}=K(\bar{\theta }-\theta )+I_{q}\int \left(\bar{\theta }-\theta )+D(\dot{\bar{\theta }}-\dot{\theta }\right),$$

where I(θ) is the soft leg’s inertia term, and $C(\theta ,\dot{\theta })$ collects Coriolis and centrifugal terms. K and D are the leg’s bending stiffness and damping, respectively. The force $\tau_{d}$ represents nonmodeled dynamics and external disturbances, including the ground reaction forces, buoyancy, and hydraulic resistance. The integral action is included to compensate for mismatches between the real system and the approximated model considered here. The constant $I_{q}$ is the gain of the integral action, which needs to be tuned in the proposed model. $\bar{\theta }$ indicates the desired parameter. Especially when the bending angle is small, and the bending deformation speed is low, $C(\theta ,\dot{\theta })$ can be omitted. Undoubtedly, the proposed method is obtained in feedback at the price of stiffening the soft robot. Actually, the walking locomotion of a fully soft robot is highly nonlinear and full of many uncertainties, belonging to the class of imperfect systems [25]. Achieving smart control of soft robots based on excitation rather than suppression of hidden dynamics is still a highly open issue. The refined theoretical model of leg’s bending dynamics and the whole-body motion control method can be further discussed on the basis of Section 2 in future extensions of this work.

### 3.3. Locomotion Experiments of the Soft Voxel-Based Robot

In this study, experiments were conducted on the presented soft voxel-based robot in an 800 × 450 × 450 mm water tank. Upon immersion, the soft voxel-based robot experiences gradual sinking due to gravitational forces. However, its hollow structure minimizes water resistance, facilitating relatively unhindered movement. The buoyancy of water uniformly supports the soft voxel-based robot, thereby reducing limb load and enhancing overall locomotion efficiency.

**Figure 8:**
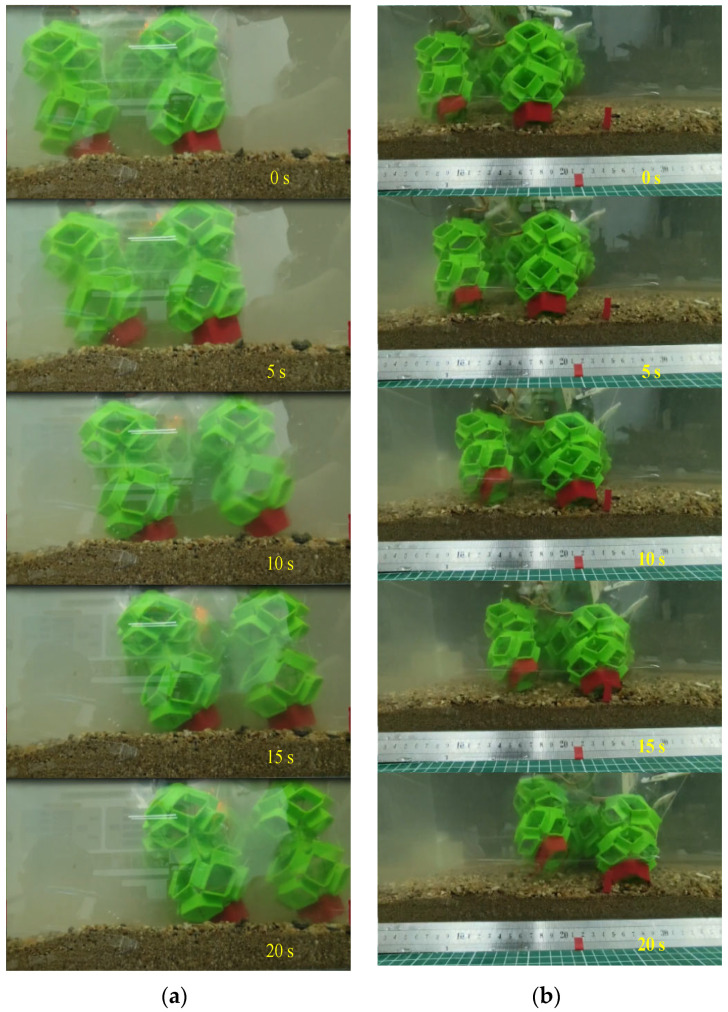
Underwater walking and crawling of the presented soft voxel-based robot on the artificial seabed. (**a**) Underwater walking on the artificial seabed; (**b**) underwater crawling on the artificial seabed.

**Figure 9:**
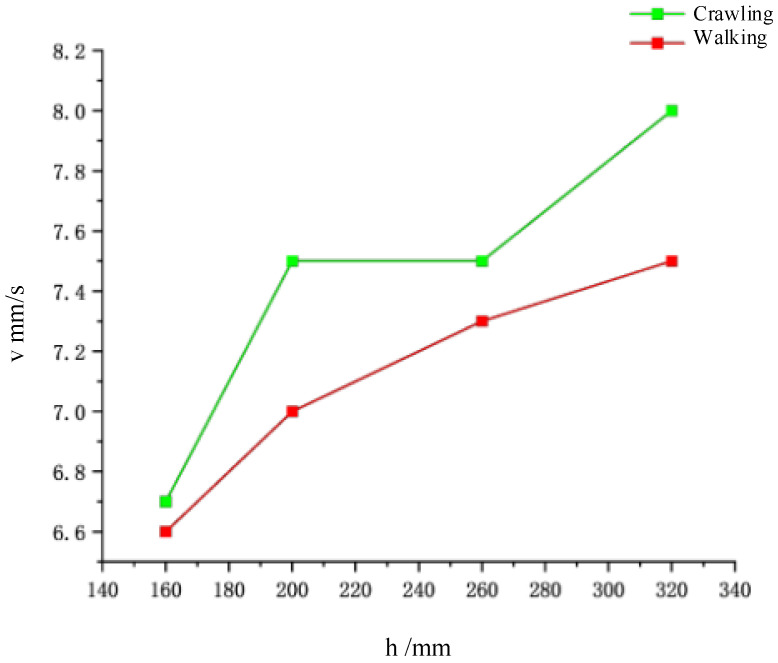
Underwater locomotion speeds of the presented soft voxel-based robot.

As illustrated in Figure 8, crawling and walking experiments were performed at varying water depths of 160 mm, 200 mm, 260 mm, and 320 mm. Experiments with the robot underscore that modulating stiffness improves locomotion performance based on the fluid content in a soil-like terrain. It was found that higher stiffness limbs allowed the robot to locomote fastest in sandy substrates and soft soils. As shown in Figure 9, the analysis revealed that at approximately a depth of 160 mm, the robot achieved speeds of approximately 6.6 mm/s for both crawling and walking. At greater depths, crawling speed increased to about 7.5 mm/s, while walking speed maintained around 7.3 mm/s.

The necessity of dealing with surges, currents, and inherent traction problems are the primary factors differentiating underwater walking from terrestrial walking. To replicate real-world underwater conditions, experiments with artificial waves were conducted. Results indicated speeds of 6.3 mm/s for underwater walking and 4.3 mm/s for crawling under surge conditions. Notably, when the surge direction aligned with the robot’s movement, the speed increased, whereas opposing directions reduced the speed. This implies how water flow can both facilitate and impede robot movement, suggesting potential improvements in underwater locomotion efficiency through mechanisms that harness water flow.

**Figure 10:**
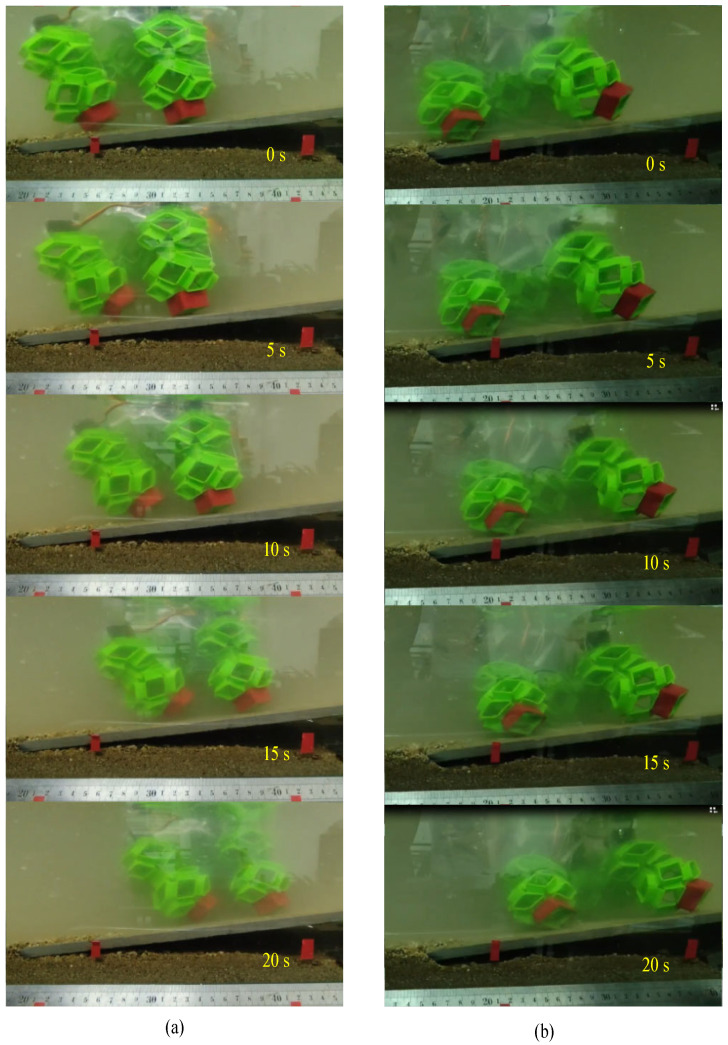
Underwater walking and crawling on a slope with the soft voxel-based robot. (**a**) Underwater walking on the slope; (**b**) underwater crawling on the slope.

In recent years, the transitional zones between ocean and land have garnered increasing attention in fields such as scientific exploration, environmental monitoring, military reconnaissance, and underwater resource survey and development. This has driven advancement in amphibious robotics technology. In the littoral zone, obstacles such as rocks, shoals, uneven slopes, dense algal beds, and reefs pose challenges that an amphibious robot may encounter and need to navigate. To explore the adaptability of the soft voxel-based robot in the transition between water and land, we conducted experiments focusing on its gradeability. As illustrated in Figure 10, at an underwater slope angle of 15°, the robot demonstrated smooth gradeability in both standing and crawling configurations. Throughout the climbing process, the robot maintained a stable body posture without noticeable shaking, tilting, or slipping. To determine the gradeability limit, we increased the underwater slope angle to 20°. Results showed moderate shaking during standing climbing and slight shaking during crawling. Further elevating the slope to 25° resulted in increased shaking and slipping for both body states of the robot. Thus, based on experimentation, the gradeability of the soft quadruped robot is limited to 20° underwater. These experiments illustrate that the soft quadruped robot possesses a notable gradeability underwater. Leveraging water buoyancy effectively reduces the robot’s weight, enabling it to navigate gently sloping underwater terrains and negotiate small obstacles like sand and stone collapses with ease.

**Figure 11:**
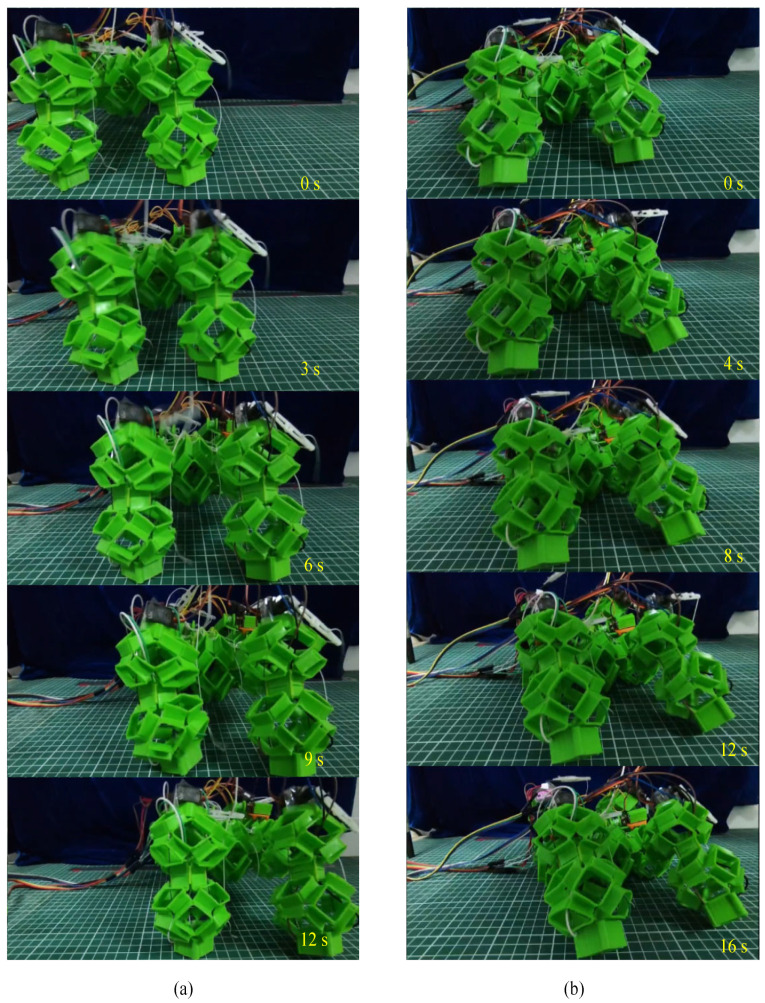
Walking and crawling on a flat terrain with the presented soft voxel-based robot. (**a**) Walking on the flat ground; (**b**) crawling on the flat ground.

**Figure 12:**
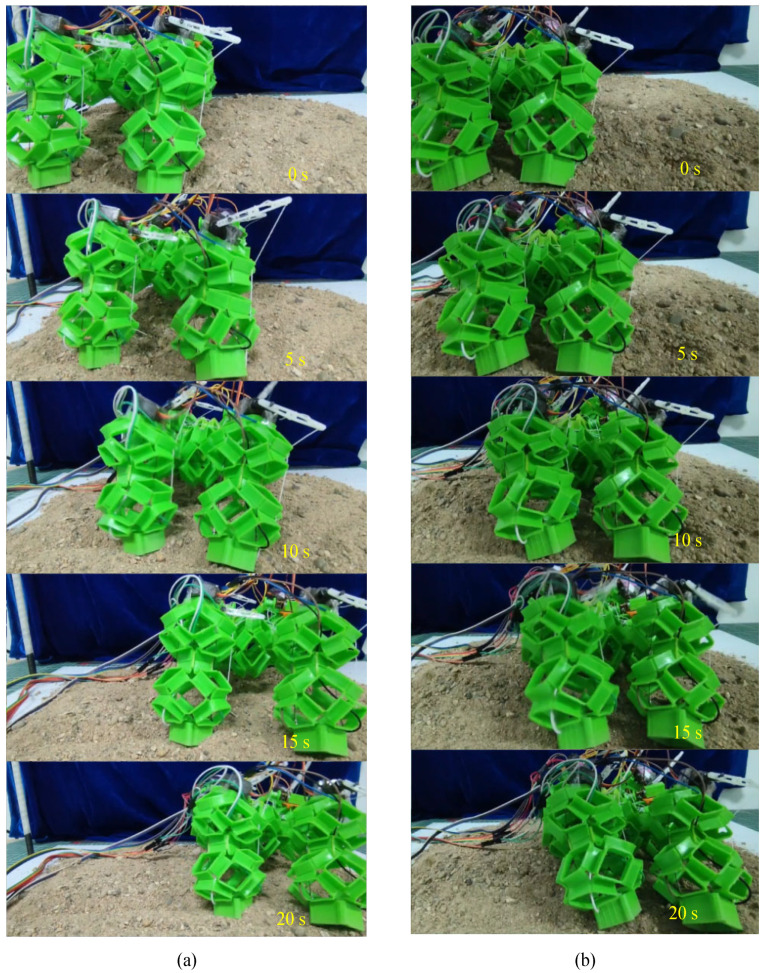
Walking and crawling on uneven sand with the presented soft voxel-based robot. (**a**) Walking on the uneven sand; (**b**) crawling on the uneven sand.

After the experiments of walking and crawling in water, we further explored the terrestrial mobility of the soft voxel-based robot. As illustrated in Figure 11 and Figure 12, we designed two distinct experimental scenarios: flat terrain and uneven sand surface. In the flat terrain experiment, the robot performed walking and crawling maneuvers on a horizontally positioned pad marked with scales. We quantified the average walking speed by measuring the linear distance covered by the robot over a specified duration. For the uneven sand scenario, a layer of sand with a thickness exceeding 20 mm was evenly spread over the horizontal surface. This prevented direct contact between the robot’s limbs and the underlying plane. While standing and walking on the sand, the robot’s limbs naturally sank due to its weight, yet the hollow structure of its cells allowed the foot’s lower end to embed slightly into the sand, enhancing overall stability. However, during crawling, this hollow structure intensified the interaction between the robot’s body and the sand, resulting in decreased movement speed. The experimental results show that the walking speed and crawling speed of the soft robot are 11 mm/s and 10 mm/s on flat terrain and 9.5 mm/s and 7 mm/s on soft sand. The voxel-type soft amphibious robot exhibits low structural rigidity, which does not hinder its movement underwater due to buoyancy. Basically, the critical fact that water is 800 times denser and 55 times more viscous than air dictates the divergent mechanisms of animal locomotion in each medium. On land, terrestrial animals move through the air where gravitational forces are predominant, necessitating support of the animal’s weight by either rigid or hydrostatic skeletons. For this reason, the absence of buoyancy requires enhanced structural rigidity in the robot’s legs and body cells. To address this, shape memory alloy (SMA) is incorporated to augment stiffness. SMA can dynamically adjust its stiffness through electrical activation and heating mechanisms.

**Figure 13:**
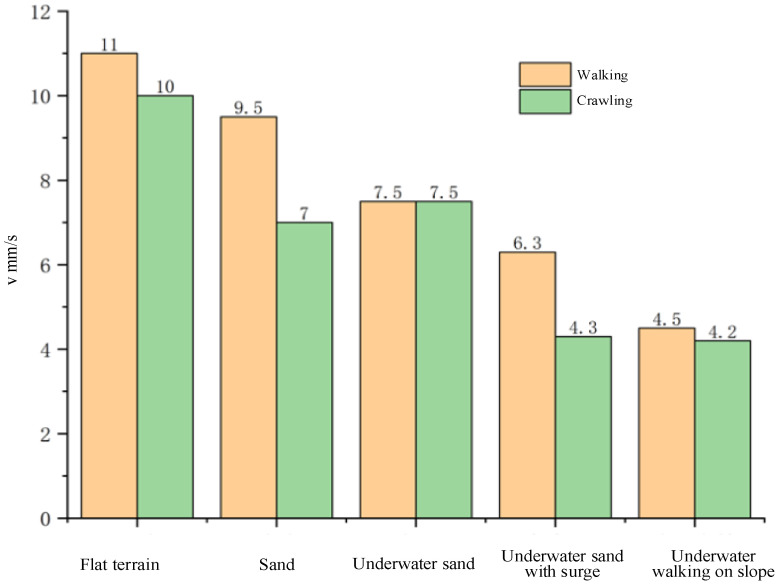
Walking and crawling speeds on various terrains with the soft voxel-based robot.

As shown in Figure 13, the soft voxel-based robot shows good mobility in underwater environments and on flat or sand terrains. Whether in aquatic or terrestrial environments, the robot achieves higher speeds on flat surfaces compared to sandy terrain. Legged robots are primarily designed for highly rugged and irregular terrains. The lack of speed can be attributed to the dissipative effects of soft materials and the low force density of soft actuators relative to motors used in rigid amphibious robots. In addition, due to their propulsion mechanisms relying solely on a single set of multilegged structures, they are capable only of crawling on land and along underwater seabeds or riverbeds, with limited swimming capabilities in the water.

### 3.4. Locomotion Capability Assessment of the Soft Voxel-Based Robot

The locomotion capability is also a comprehensive performance metric for amphibious robots. To evaluate the locomotion capabilities of the soft quadruped robot, we adopt the amphibious robot locomotion capability evaluation coefficient [21], i.e., the locomotion capability of the amphibious robot (LCAR). LCAR is defined as the ratio of the robot’s running speed to its own weight, denoted by M. The robot’s running speed can be expressed in meters per second, denoted by m/s, or as body lengths per second, denoted by BL/s, denoted by $v_{meter}$ and $v_{body}$, respectively.

(10) $$LCAR=\frac{v_{meter}}{M}=\frac{v_{body}}{M}.$$

Based on calculations, the LCAR for crawling on sand is 0.015, while for other scenarios, it remains around 0.02. When considering the body dimensions of the robot as 154.77 mm in length and 206.36 mm in width, sand crawling yields 0.045 BL/s and an LCAR of 0.101, whereas other scenarios maintain around 0.065 BL/s with an LCAR of 0.146.

For underwater crawling at a speed of 7.5 mm/s and walking at 7.3mm/s, with a mass of 447 g, converted into consistent units, the LCAR values of 0.017 and 0.016 are derived, respectively. When considering body length per second, the speed is 0.05 BL/s, resulting in LCAR values of 0.108 for crawling and 0.106 for walking. These calculations confirm that the robot’s locomotion capabilities fall within the range of 0 to 0.3 for LCAR, accommodating different volumes and masses of robots.

Further, as shown in Table 1, we compare the presented soft quadruped robot with robots developed by other researchers. First, the overall body of our robot is fully hollow, which can more easily adapt to changes in pressure encountered at different depths in the deep sea. Unlike closed fluid chambers that may require specific pressure regulation mechanisms, the hollow structure can deform slightly to adjust to varying external pressures without risking leakage or structural damage. Second, unlike some reference robots that need tethers, our robot works without tethers. This autonomy can provide freedom and flexibility of operation, especially in deep water or deep-sea environments where tethering may be impractical or limited.

## 4. Conclusions

Designing the structure of amphibious robots presents significant challenges, given the stringent demands for flexibility, stability, integrity, and adaptability in trans-media environments. This research work demonstrates a novel voxel-type soft amphibious robot based on an assemblage of origami flexiballs. Theoretically, the geometric model and elastic constitutive models of the origami flexiball are established to depict its complex deformation mechanisms. Encouragingly, we connect fourteen flexible balls into an assemblage to create a voxel-type soft amphibious quadruped robot. Experimental work indicates that the presented robot can walk or crawl in underwater environments and on flat or sand terrains. To wrap up, the voxel-type soft amphibious quadruped robot has the following advantages:

- Easy to manufacture. The 3D printing method with accessible soft elastic materials enables the voxel-type soft robot to be fabricated rapidly and at a low cost. The overall structure has no complex parts, and no complicated assembly or die preparation is required.
- Adaptability to multiple environments. One of the primary advantages is its ability to move both underwater and on land without the need for complex shape-shifting mechanisms. Their ability to seamlessly transition between aquatic and terrestrial environments makes them versatile for a wide range of applications, from marine exploration to terrestrial tasks. In particular, the robot’s origami polyhedra-induced hollow structure enables it to naturally adapt to underwater conditions, including variations in hydrostatic pressure and water currents. This adaptation enhances its stability and performance during underwater operations.
- Compact and modular design. Voxel-type soft robots are typically designed with modular components that enable compact storage and deployment. This modularity facilitates easy maintenance and reconfiguration for different mission requirements, enhancing their overall utility.
- Innovative design potential. By leveraging metamaterial features embodied in the origami polyhedra, these robots contribute an innovative approach to soft robotics. They demonstrate how geometrically inspired designs can achieve complex functionalities while maintaining simplicity and reliability in operation.

Admittedly, the current project has lots of room for improvement, in particular in terms of perception and maneuverability. A pivotal area for improvement lies in mobility, necessitating precise refinements in limb design and propulsion systems to achieve heightened agility and maneuverability across aquatic and terrestrial terrains. Integral to this advancement is the integration of sophisticated sensing and control systems, which are essential for robust environmental data acquisition and autonomous adaptation to dynamic conditions. This includes deploying advanced sensors for precise navigation, object detection, and comprehensive environmental monitoring.

Ensuring durability under harsh conditions, such as deep-sea pressures or rugged landscapes, requires the exploration of resilient materials and protective coatings to guarantee sustained reliability over extended operational periods. Expanding the robot’s capabilities for manipulation tasks, encompassing object handling and tool deployment both underwater and on land, further enhances its utility across diverse applications—from maintenance operations to scientific sampling endeavors. In addition, soft robots also require flexible, lightweight power sources with high energy density relative to their size, and weight is crucial for maximizing operational endurance.

A critical frontier in advancing these robots lies in autonomous decision-making capabilities. Employing sophisticated algorithms for efficient path planning, obstacle avoidance, and task prioritization minimizes reliance on human intervention, thereby enhancing operational autonomy and efficiency. By prioritizing these interconnected advancements, we can propel voxel-type soft amphibious robots toward broader applications in fields like marine exploration, infrastructure maintenance, and scientific research. This approach not only fosters innovation but also facilitates adaptation to varied and challenging environments, underscoring the transformative potential of these technologies in shaping the future of robotics.

## Figures and Tables

**Table 1:** Comparison of different soft quadruped amphibious robots in navigation capacity.

Robot	Year	Actuation	Speed (BL/s)	Terrain Adaptability	Tethering
The presented robot	2024	Rope-motor	⮚ 0.07(Ground walking) ⮚ 0.05(Underwater crawling)	⮚ Slope ⮚ Sand ⮚ Water ⮚ Ground	No
Ref. [4]	2023	Pneumatic	⮚ 0.13(Land crawling) ⮚ 0.23(Swimming)	⮚ Gravel ⮚ Grass road ⮚ Water	Yes
Ref. [26]	2022	Pneumatic	⮚ 0.97(Land crawling)	⮚ Water ⮚ Small hill ⮚ Gravel ⮚ Road ⮚ Sand	Yes
Ref. [27]	2022	Motor	⮚ 0.08(Land crawling)	⮚ Ground ⮚ Underwater	Yes
Ref. [28]	2017	Pneumatic	\	⮚ Slope ⮚ Sand ⮚ Water	Yes

## Data Availability

All data generated or analyzed during this study are included in this article.
